# Influence of Culture Conditions and Medium Compositions on the Production of Bacteriocin-Like Inhibitory Substances by *Lactococcus lactis* Gh1

**DOI:** 10.3390/microorganisms8101454

**Published:** 2020-09-23

**Authors:** Roslina Jawan, Sahar Abbasiliasi, Joo Shun Tan, Shuhaimi Mustafa, Murni Halim, Arbakariya B. Ariff

**Affiliations:** 1 Bioprocessing and Biomanufacturing Research Centre, Faculty of Biotechnology and Biomolecular Sciences, Universiti Putra Malaysia, Serdang 43400, Malaysia; roslinaj@ums.edu.my (R.J.); murnihalim@upm.edu.my (M.H.); 2 Biotechnology Programme, Faculty of Science and Natural Resources, Universiti Malaysia Sabah, Kota Kinabalu 88400, Malaysia; 3 Halal Products Research Institute, Universiti Putra Malaysia, Serdang 43400, Malaysia; sahar@upm.edu.my (S.A.); shuhaimimustafa@upm.edu.my (S.M.); 4 Bioprocess Technology, School of Industrial Technology, Universiti Sains Malaysia, Gelugor 11800, Malaysia; jooshun@usm.my; 5 Department of Microbiology, Faculty of Biotechnology and Biomolecular Sciences, Universiti Putra Malaysia, Serdang 43400, Malaysia; 6 Department of Bioprocess Technology, Faculty of Biotechnology and Biomolecular Sciences, Universiti Putra Malaysia, Serdang 43400, Malaysia

**Keywords:** fermentation, *Lactococcus lactis*, bacteriocin, culture conditions, medium compositions

## Abstract

Antibacterial peptides or bacteriocins produced by many strains of lactic acid bacteria have been used as food preservatives for many years without any known adverse effects. Bacteriocin titres can be modified by altering the physiological and nutritional factors of the producing bacterium to improve the production in terms of yield and productivity. The effects of culture conditions (initial pH, inoculum age and inoculum size) and medium compositions (organic and inorganic nitrogen sources; carbon sources) were assessed for the production of bacteriocin-like inhibitory substances (BLIS) by *Lactococcus lactis* Gh1 in shake flask cultures. An inoculum of the mid-exponential phase culture at 1% (*v*/*v*) was the optimal age and size, while initial pH of culture media at alkaline and acidic state did not show a significant impact on BLIS secretion. Organic nitrogen sources were more favourable for BLIS production compared to inorganic sources. Production of BLIS by *L. lactis* Gh1 in soytone was 1.28-times higher as compared to that of organic nitrogen sources ((NH_4_)2SO_4_). The highest cell concentration (X_mX_ = 0.69 ± 0.026 g·L^−1^) and specific growth rate (μ_max_ = 0.14 h^−1^) were also observed in cultivation using soytone. By replacing carbon sources with fructose, BLIS production was increased up to 34.94% compared to BHI medium, which gave the biomass cell concentration and specific growth rate of 0.66 ± 0.002 g·L^−1^ and 0.11 h^−1^, respectively. It can be concluded that the fermentation factors have pronounced influences on the growth of *L. lactis* Gh1 and BLIS production. Results from this study could be used for subsequent application in process design and optimisation for improving BLIS production by *L. lactis* Gh1 at larger scale.

## 1. Introduction

The use of antimicrobials in shelf life enhancement of foods is a new branch of science. Bio-preservation is a technique used for extending the shelf life of food using natural or controlled microbiota or antimicrobials. The fermentation products as well as beneficial bacteria are generally selected in this process to control spoilage and render pathogen inactive. The special interest organism or central organism used for this purpose is lactic acid bacteria (LAB) and their metabolites [1]. LAB are capable of producing various antimicrobial compounds such as organic acids (lactic acid and acetic acid), diacetyl, ethanol, hydrogen peroxide, reuterin, acetaldehyde, acetoine, carbon dioxide and bacteriocins during fermentation processes [2].

LAB bacteriocins are considered good bio-preservative agents due to their non-toxic, non-immunogenic and thermo-resistance characteristics as well as broad bactericidal activity. These bacteriocins are most effective against Gram-positive bacteria and some damaged, Gram-negative bacteria including various pathogens such as *Listeria monocytogenes*, *Bacillus cereus*, *Staphylococcus aureus*, *Salmonella* in foods [1,3]. The use of bacteriocins as natural food preservatives fulfils consumer demands for high quality and safe foods without the use of chemical preservatives [4]. For the past years, bacteriocins have attracted considerable interest for their use as safe food preservatives since they are easily digested by the human gastrointestinal tract [5]. Several reports have showed that antimicrobial metabolites produced by *Lactococcus lactis* exhibit broad inhibitory property towards species that are closely related to LAB and other unrelated spoilage and pathogenic bacteria [6,7].

Recent research indicated that LAB and their natural products can offer promising opportunities for the development of efficient food bio preservation strategies [8,9,10]. The potential applications of bacteriocins from LAB as bio-preservatives for the inhibition of proliferation of *L. monocytogenes* in foods have been reported [11,12,13,14]. Generally, most LAB-bacteriocins act on pathogen cells by destabilisation and permeabilisation of the cytoplasmic membrane through the formation of transitory poration complexes or ionic channels that cause the reduction or dissipation of the proton motive force (PMF). Bacteriocins producing LAB strains protect themselves against the toxicity of their own bacteriocins by the expression of a specific immunity protein that is generally encoded in the bacteriocin operon namely induction factor (IF), histidine protein kinase (HPK) and a response regulator (RR) [15]. As food bio-preservative, bacteriocin could be used either as a food additive or through the application of bacteriocin-producing culture. However, the latter would necessitate the optimisation of specific fermentation conditions [16]. Formulation of the growth medium is an important factor that needs to be considered in producing any microbial products involving fermentation processes. On the other hand, formulation for industrial scale application should fulfil a number of criteria including cost-effectiveness, high product yield, short fermentation time and ease of downstream purification processes [17].

Specific requirements with reference to the production of bacteriocins through microbial fermentation have been reported by several studies [18,19,20]. Bacteriocin titres can be modified by altering the cultivation conditions of the producing bacterium and certain combinations of influencing factors. However, the influencing factors may be strain dependent and could vary with different types of bacteriocin. The optimal design of culture media is an important aspect to be considered when developing a fermentation process. The formulation of media containing complex nutrients is generally preferred for large-scale fermentations since it leads to the development of cost-effective processes that support maximum product yield. In the initial formulation of the medium in batch culture, an effort has been made to understand the best source of carbon and energy as well as the regulatory aspects of the enzyme [21].

The growth of bacteria and accumulation of their metabolites are strongly influenced by the environment and medium compositions such as culture pH, carbon and nitrogen sources, growth factors as well as minerals. It is difficult to detect these major factors and optimise them for biotechnological processes including multivariable [22]. The properties of the growth media including amino acid composition, carbon/nitrogen ratio, pH and lactose levels have a great influence on the change in biomass of the culture and the corresponding change in the level of bacteriocin production [23]. De Man, Rogosa and Sharpe (MRS) medium is usually the medium of choice for studying LAB fermentations, but it has its limitations. Using MRS broth or any commercially available laboratory-grade defined growth media for industrial production of bacteriocins would be prohibitively expensive besides involving unauthorised ingredients as food additives [24]. Therefore, the objective of the present study was to optimise the physiological (pH value, inoculum age and size) and nutritional (medium compositions) factors for improving the growth and ability of *Lactococcus lactis* Gh1 to secrete bacteriocin-like inhibitory substances (BLIS) in shake flask fermentation.

## 2. Materials and Methods

### 2.1. Materials

Culture media (Brain heart infusion (BHI), M17, MRS, tryptic soy broth (TSB) and LB media) and inorganic nitrogen sources ((NH_4_)_2_SO_4_, NH_6_PO_4_, NH_4_NO_3_ and NH_4_Cl) were purchased from Merck (Darmstadt, Germany). Carbon sources (fructose, glucose, galactose, sucrose, lactose, maltose, sorbitol and mannitol) were obtained from Fisher Chemical (Loughborough, United Kingdom), while organic nitrogen sources (yeast extract, meat extract, peptone and soytone) were from BD (Franklin Lakes, NJ, USA).

### 2.2. Microorganisms and Maintenance

A BLIS-producing *Lactococcus lactis* Gh1 isolated from a milk by-product of an Iranian traditional fermented milk was used throughout this study [25,26]. The indicator microorganism in antimicrobial activity was *Listeria monocytogenes* ATCC 15313. The stock cultures were maintained at −80 °C in MRS and BHI broth (Merck, Darmstadt, Germany), respectively, supplemented with 20% (*v*/*v*) glycerol (BDH Laboratory Supplies, Poole, UK), for use in the fermentation experiments.

### 2.3. Preparation of Inoculum and Culture Condition

The stock culture of *L. lactis* Gh1 was first revived on the BHI agar (Merck, Darmstadt, Germany) prior to the preparation of inoculum. A single colony of *L. lactis* Gh1 was cultured in 10 mL of BHI broth and incubated at 30 °C for 24 h. The 1% (*v/v*) of the culture was sub-cultured at 30 °C for 16–18 h before being used as an inoculum. The optical density (OD) of the culture at 650 nm was standardised at 1.89–2.00 (≈2.68 × 10^9^ CFU/mL) and used as an inoculum for all fermentations with the size of 1% (*v*/*v*). All experiments were conducted in 100 mL of BHI broth in 250 mL of Erlenmeyer flasks. The cultures were incubated at 30 °C in a horizontal shaker (B. Braun Biotech International, Melsungen, Germany) and agitated at 100 rpm for 16 h.

### 2.4. Factors Influencing the Production of Bacteriocin-Like Inhibitory Substances (BLIS)

In order to identify the factors that influence BLIS production, the one-factor-at-a-time (OFAT) approach was used. The selected factors were applied to the next experiment. The selection of the appropriate factors was based on the highest BLIS production.

### 2.5. Selection of Culture Media for BLIS Production

Five types of commercial culture media (MRS, M17, BHI, TSB and LB) were initially tested for production of BLIS.

### 2.6. Effects of Initial pH of Culture Media on BLIS Production

The initial pH of the selected media was adjusted to pH 2–9 by the addition of either 1.0 M of HCl or 1.0 M NaOH.

### 2.7. Effects of Inoculum Age, Size and Cultivation Conditions on BLIS Production

Based on the growth phase of *L. lactis* Gh1, three different inoculum ages namely early-exponential, late exponential and stationary phases were evaluated. The selected inoculum age with maximum BLIS secretion was added into the media at different concentrations ranging from 0.5% to 10% (*v*/*v*). The optimum inoculum size and age was incubated in condition without and with agitation at 100 rpm.

### 2.8. Screening of Medium Composition for BLIS Production

To initiate the fermentation, a 250 mL Erlenmeyer flask containing 100 mL of BHI broth was inoculated with 1% (*v*/*v*) of inoculum at mid-exponential growth phase and incubated at 30 °C on a rotary shaker, which then agitated at 100 rpm for 16–18 h. BHI medium was selected in this study as this medium promoted significant BLIS production. The composition of BHI medium is shown in Table 1.

To evaluate the effects of nitrogen sources on the production of BLIS, the nitrogen component in the BHI medium was replaced with different types of organic (yeast extract, meat extract, peptone and soytone) and inorganic ((NH_4_)_2_SO_4_, NH_6_PO_4_, NH_4_NO_3_ and NH_4_Cl) nitrogen sources. The amount of nitrogen was calculated based on the original quantity of nitrogen (N) in BHI medium. Nutrient substrate (27.5 g/L) of BHI medium is equivalent to 4.6 g of N. Therefore, nitrogen sources were prepared according to this nitrogen amount.

In the subsequent experiment to study the effects of different carbon sources namely monosaccharides (fructose, glucose, galactose), disaccharides (sucrose, lactose, maltose) and sugar alcohols (sorbitol, and mannitol) on BLIS production, soytone was used as a preferred nitrogen source.

### 2.9. Analytical Procedures

During the fermentation, samples were withdrawn at 2-h intervals and prepared for analysis. The changes in culture pH were measured using a pH meter (Mettler-Toledo, Switzerland). The culture samples were centrifuged (Eppendorf, Centrifuge 5810R, Hamburg, Germany) at 13,751× *g* for 10 min at 4 °C. The cell pellets were washed and resuspended twice with saline water (0.85%, *w*/*v* NaCl) for turbidity determination and read at 600 nm using a spectrophotometer (Biochrom, Libra S12, UK). The optical density (OD) was converted into dry cell weight (DCW) from a standard curve using an experimentally predetermined factor of 0.26 where one OD unit was equivalent to 0.26 of DCW per volume (g·L^−1^). Antibacterial activity (AU/mL) against *L. monocytogenes* ATCC 15313 was quantitatively performed by the agar well diffusion method as described by Abbasiliasi et al. [25]. Briefly, the culture of *L. lactis* Gh1 was centrifuged (Eppendorf, Centrifuge 5810R, Hamburg, Germany) at 13,751× *g* for 10 min at 4 °C. The supernatant (100 μL) was put in 6-mm agar plate wells that were previously seeded (1%, *v*/*v*) with an active-growing *L. monocytogenes* ATCC 15313. The plates were then placed at 4 °C for well diffusion of the sample into the agar media for 2 h prior to incubation at 37 °C. After 24 h of incubation, the inhibition zone of the supernatant against the indicator bacteria was measured using electronic calliper (in mm). The quantification of the antimicrobial activity (AU/mL) of BLIS was calculated using Equation (1):

(1) $$\mathrm{BLIS}\;\mathrm{activity}\;(\frac{AU}{mL})\;=\;\frac{A_{Z}-A_{w}}{V}$$

where, A_z_ = clear zone area (mm^2^), A_w_ = well area (mm^2^), *V* = volume of sample (mL).

### 2.10. Statistical Analysis

Analysis of variance (ANOVA) for mean data was performed by IBM SPSS Statistics 25 software. Duncan multiple range test was used to determine the significance among the treatments means with significant level at *p* < 0.05.

## 3. Results

### 3.1. Effects of Culture Media on the Production of BLIS

Among the culture media tested in this study, there were no significant differences (*p* < 0.05) in BLIS production between M17, BHI and TSB (Table 2). However, the highest BLIS production (672.86 ± 24.76) and μ_max_ (0.15 ± 0.0126 h^−1^) were recorded in BHI. MRS medium promoted better cell growth (0.49 ± 0.002 g·L^−1^) compared to other media. The BLIS production was shown by the inhibition zones of *L. lactis* Gh1 supernatant against the *L. monocytogenes* ATCC 15313 in the antimicrobial assay. The increase in size of the inhibition zones was related to the inhibitory effect of the *L. lactis* Gh1.

**Figure 1:**
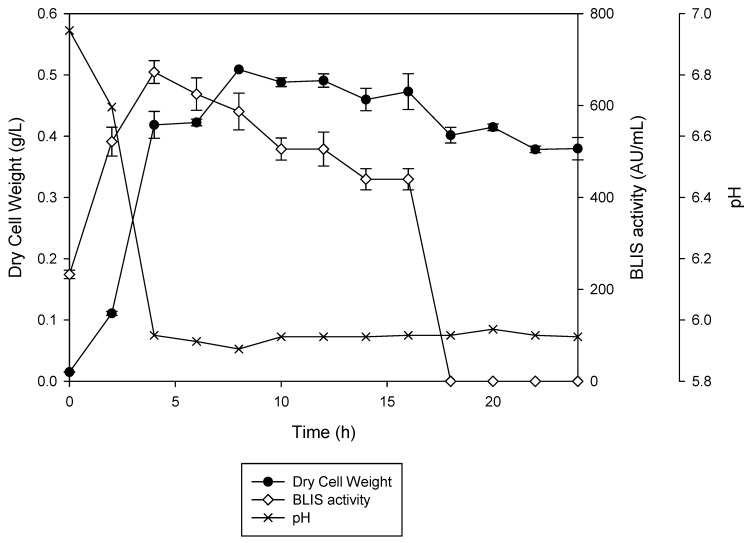
Growth profile of *L. lactis* Gh1 in Brain heart infusion (BHI) broth.

The time course of BLIS production by *L. lactis* Gh1 is shown in Figure 1. The highest BLIS activity was reached at the late-exponential phase (4 h) and decreased afterwards when the cells entered the stationary phase. The BLIS activity was not detected at 18 h of incubation. The pH was decreased rapidly during the maximum production of BLIS and remained constant up to the end of incubation time. The specified pattern of cell growth and BLIS production indicated that the produced BLIS was a primary metabolite. BLIS production was reduced once cells entered the stationary phase. Since the use of BHI enhanced the growth of *L. lactis* Gh1 and BLIS production, this medium was selected for subsequent use in the statistical optimisation for further improving the fermentation performance.

### 3.2. Effects of Initial pH on BLIS Production

As shown in Table 3, the initial pH of the culture among the alkaline pH (7–9) did not give a significant difference (*p* < 0.05) on BLIS production by *L. lactis* Gh1. However, BLIS secretion started to decrease at pH lower than 6, whereas BLIS secretion and cell growth were suppressed at pH lower than 4. The BHI medium without pH alteration (pH: 7.04) recorded the highest BLIS production (599.45 ± 11.77 AU. mL^−1^), cell concentration (0.36 ± 0.013 g L^−1^) and specific growth rate (0.104 ± 0.0025 h^−1^). The fermentation time to attain the maximum BLIS activity (P_mX_) was the same (6 h) for all initial culture pH tested in this study.

### 3.3. Effects of Inoculum Age, Size and Sub-Culture Frequency on BLIS Production

Growth of *L. lactis* Gh1 and BLIS production at different inoculum ages is shown in Table 4. Inoculum at mid-exponential growth phase recorded the highest BLIS production (665.96 ± 1.83 AU. mL^−1^) followed by the activity at stationary (627.98 ± 16.74 AU. mL^−1^) and late-exponential (615.73 ± 5.34 AU. mL^−1^) growth phases. In fermentation with inoculum from mid-exponential phase, cells tend to produce BLIS without growing

The effects of inoculum size on growth of *L. lactis* Gh1 and BLIS production is shown in Table 5. The results demonstrated that the size of inoculum has a strong relation with the production of BLIS. The fermentation with inoculum size of 1% (*v/v*) produced the highest BLIS compared to that with high inoculum size (>2% *v/v*). BLIS secretion was the lowest at the highest inoculum size (10%, *v/v*) so far studied. The inoculum size ranging from 4% (*v/v*) to 10% (*v/v*) produced more cells than BLIS production. The use of inoculum at a stationary growth phase and with the lowest or highest size influenced the duration of the lag phase, which gave a longer time to reach to P_max_ after 8–10 h of fermentation.

BLIS production by *L. lactis* Gh1 was also influenced by the frequency in the sub-culturing of inoculum (Table 6). Good cell growth with enhanced BLIS productivity was observed in fermentation with inoculum sub-cultured for two times, which was two times higher (728.83 ± 37.80 AU. mL^−1^) compared to that obtained in fermentation with a single time sub-cultured inoculum (553.45 ± 4.58 AU. mL^−1^) with shorter lag phase length.

### 3.4. Effects of Different Organic and Inorganic Nitrogen Sources on BLIS Production

The effects of organic and inorganic nitrogen sources on growth of *L. lactis* Gh1 and production of BLIS are shown in Table 7. Organic nitrogen sources were more favourable for BLIS production compared to inorganic sources. *L. lactis* Gh1 grown in soytone-supplemented medium produced the maximum BLIS production (764.71 ± 15.15 AU. mL^−1^) and maximum cell (0.69 ± 0.026 g L^−1^) significantly higher (*p* < 0.5) than those obtained by other organic (yeast extract, meat extract, peptone, tryptone, soytone) or inorganic ((NH_4_)_2_SO_4_, NH_4_NO_3_, NH_6_PO_4_, NH_4_Cl) nitrogen sources. The replacement of nitrogen source of BHI medium with soytone resulted in shorter lag phase length and time (4 h) in producing maximum BLIS compared to BHI and inorganic nitrogen sources (6 h).

### 3.5. Effects of Different Types of Carbon Source on BLIS Production

Effects of different types of carbon sources on BLIS production by *L. lactis* Gh1 are shown in Table 8. BLIS production was the highest (707.43 ± 16.83 AU. mL^−1^) in fermentation using fructose compared to other carbon sources (galactose, glucose, sucrose, lactose, maltose, mannitol and sorbitol) tested in this study. In modified BHI medium, the replacement of carbon and nitrogen sources with fructose and soytone increased the BLIS production up to 34.94% compared to commercial BHI. The role of carbon sources was unenviable as long as the nitrogen was added into the media since the BLIS production (634.82 ± 26.40 AU. mL^−1^) was still detected in the control treatment (without carbon sources). In addition, BLIS production in control was higher compared to that of BHI medium indicating the beneficial effect of soytone in supplying other minerals needed by *L. lactis* Gh1 than nitrogen components.

## 4. Discussion

The production of BLIS is reliant on the type of culture media and the composition. The inverse relationship between cell growth and bacteriocin production in this study was supported by Ünlü, Nielsen and Ionita [27] who stated that the good bacterial growth does not guarantee good bacteriocin production although bacteriocin production is associated with bacterial growth. BHI is one of the complex media for the cultivation of LAB available in today’s market beside de Man Rogosa and Sharpe (MRS), NaLa (sodium lactate), M17 and trypticase soy broth yeast extract (TSBYE) [28]. However, contradicting the reported literature, BHI was rarely preferred as a good media for bacteriocin production due to low productivity [17]. To date, there were not many findings highlighting the suitability of BHI in the production of BLIS from LAB. In agreement with many reports [29,30,31,32] medium composition greatly influenced BLIS production of LAB. Sodium chloride (NaCl) was one of the major components in the medium used in this study (Table 1), which enhanced and stabilised BLIS secretion [33].

The specified pattern of cell growth and BLIS production in the current study indicated that the produced BLIS was a primary metabolite. This finding is in agreement with Taheri, Samadi, Ehsani, Khoshayand and Jamalifar [34] as well as Lv, Zhang and Cong [35]. BLIS production was reduced once cells entered the stationary phase, which might be due to the proteolytic degradation during lysis, aggregation and/or adsorption of bacteriocins on the cell wall of the producing microorganisms [36]. The inconsistencies of bacteriocin production kinetic, either growth associated or non-growth associated, are related to pH dependent phenomena such as the adsorption of bacteriocins onto cell surfaces and/or the post-translational processing of the pre-peptides to active forms [37,38]. Production of bacteriocin by LAB usually follows primary metabolite growth-associated kinetics in which the production occurs during exponential growth phase and ceases once stationary phase is reached [39]. This is however not always the case and the relationship between bacteriocin production and growth are strain dependent [40]. In some cases, a correlation exists between peptide- and biomass production [41,42] while in other cases, bacteriocin production only starts at the beginning of stationary phase [39,43,44,45]. Recently, the BLIS produced by *Lactococcus lactis* Gh1 has been characterised [26]. The activity of BLIS produced by *L. lactis* Gh1 did not change with changes in pH from pH 4.36 to pH 8, which confirmed the proteinaceous nature of BLIS as antimicrobial substance.

Specific requirements with reference to the production of bacteriocins by LAB have been reported. The type or composition of culture medium especially nitrogen and carbon source greatly influenced bacteriocin production [36]. Lowering the amount of the organic nitrogen sources in the medium while keeping the nisin yield constant is advantageous not only for bacteriocin purification, but also for lowering the production cost [28]. All of these media are good for neutralising lactic acid and improving cell growth, but do not consider the accumulation of bacteriocin and high content of nitrogen sources especially proteins and peptides that may bring about the difficulties of bacteriocin purification. Since the use of BHI enhanced the growth of *L. lactis* Gh1 and BLIS production, this medium was selected for subsequent use in the statistical optimisation for further improving fermentation performance.

BLIS secretion started to decrease at pH lower than 6, whereas BLIS secretion and cell growth were suppressed at pH lower than 4. In general, culture pH greatly influences cell growth and bacteriocin production by aggregation, adsorption of bacteriocin to the cells and/or proteolytic degradation of bacteriocin [17,46]. The dependency of bacteriocin production on culture pH indicates that pH could regulate the expression of biosynthetic genes similarly observed for several classes of genes [47]. Initial pH level of culture medium is one of the key factors influencing the growth of the bacteriocinogenic LAB strains and the adsorption of bacteriocins onto the cell wall of the producing microorganisms [48]. Reduced BLIS production at pH below 6 as observed in this study is in agreement with the report by Yang et al. [48] who claimed that BLIS was maximally adsorbed to LAB cells at pH ranging from 6.0 to 4.0. Whereas the stunted cell growth and BLIS production at pH lower than 4 have been reported by many researchers [32,48,49]. Nutrient transport, which is a pH-function, may be one of the growth rate limiting actions in LAB. Therefore, the failure to grow at acidic pH is likely to be caused by a limitation of cytoplasmic processes (acidification of the cytoplasm and collapse of the motive force) [50,51,52].

Inoculum at mid-exponential growth phase recorded the highest BLIS production. In batch fermentation, the highest growth rate is at mid-exponential phase. The rate of cell growth in the culture is proportionate to the number of cells present at any given time during exponential growth phase [53], an important role in bacteriocin production where the optimum inocula size is favourable for the highest productivity [54]. The results demonstrate a strong relation of the inoculum size with the production of BLIS. Addition of 1% (*v*/*v*) of inoculum was intensively applied in inoculum preparation of LAB as reported previously [39,55]. Optimal size of inocula may vary from strain producers due to their cell proliferation rate, ability to metabolise medium, mass transfers, medium size and nutrient composition. However, minimum inocula size is preferred due to easier inoculation in large scale fermentation [54]. BLIS secretion was the lowest at the highest inoculum size (10%, *v*/*v*) so far studied. The inoculum size ranging from 4% (*v*/*v*) to 10% (*v*/*v*) produced more cells than BLIS production. High inoculum size has been attributed to a substantial reduction in oxygen tension at high bacterial densities [56]. The use of inoculum at a stationary growth phase and with the lowest or highest size influenced the duration of the lag phase, which gave a longer time to reach to P_max_ after 8–10 h of fermentation.

Organic nitrogen sources were more favourable for BLIS production as compared to inorganic sources. *L. lactis* Gh1 grown in soytone-supplemented medium produced maximum BLIS production and cell significantly higher than those obtained by other organic or inorganic nitrogen sources. The replacement of nitrogen source of BHI medium with soytone also resulted in shorter lag phase length and time (4 h) for producing maximum BLIS compared to that with BHI and inorganic nitrogen sources (6 h). This finding is in line with that by Ramachandran et al. [57] who concluded that organic nitrogen gave higher bacteriocin yield compared to inorganic nitrogen sources. Lechiancole et al. [58] stated that the growth and bacteriocin production of *L. sakei* were markedly improved with the replacement of tryptone with bacteriological peptone or soytone. Soytone is rich in minerals such as magnesium, potassium, sodium, chloride, sulphate, phosphate as well as free amino acids such as alanine, arginine, asparagine, and aspartic acid. The growth of *L. lactis* Gh1 and BLIS production was supressed in the absence of nitrogen sources in the culture medium, indicating the crucial function of nitrogen supplementation in supporting the cell growth. Lactococcal strains are nutritionally fastidious microorganisms, in which cell growth and bacteriocin production are influenced by a rich medium with organic nitrogen source [36]. Nitrogen sources are required for all processes involving biological growth especially with reference to synthesis of cellular protein and nucleic acid production as well as for bacterial metabolism [17]. The ability of LAB to metabolise different carbon sources is based on the specific activities of the enzymes involved in carbohydrate degradation; for example, amylolytic LAB have the ability to secrete amylase, which hydrolyse starch to fermentable sugars [59]. Osmotic stress, which increases the energy demand, apparently reduces the maximum secretion of bacteriocin, indicating that the energy is required in excess for the synthesis [17]. In bacteriocin fermentation, glucose is the preferred carbon source to stimulate bacteriocin production. Many researchers demonstrated that high bacteriocin yield was associated with the presence of glucose in growth medium and no other monosaccharides [60]. The highest bacteriocin production was recorded with the addition of glucose, maltose, lactose, and sucrose [17]. In contrary to the results of this study, fructose was found to be the most appropriate carbon source for BLIS production by *L. lactis* Gh1. Fructose was not preferentially metabolised compared to other carbon sources for bacteriocin production [61,62]. Stolz et al. [63] reported the use of fructose as an energy source by *L. reuteri* LTH 3120 and *L. amylovorus* LTH 3122. Fructose was also considered as the preferred energy source for maintenance, especially at temperatures greater than 34 °C in which fructose was converted by *L. amylovorus* DCE 471 at a faster rate than maltose [64].

Generally, bacteriocin production is a growth associated process. Higher cell density accumulates more inducer peptides that function in the quorum sensing regulations to induce bacteriocin production [65]. However, these facts are inconsistent with the findings in this study. The selected parameter with the highest BLIS production exhibited contradictory results with the cell growth. This observation was found in most fermentation factors tested in this study (media, inoculum preparation and carbon sources). From the results of this study, it can be claimed that BLIS production by *L. lactis* Gh1 did not follow the general growth-dependent bacteriocin production. Some bacteriocins are favourably produced in unfavourable growth conditions [66,67].

## 5. Conclusions

Results from this study demonstrated that the growth of *L.* *lactis* Gh1 and BLIS production were influenced by the physiological (initial pH, inoculum age and inoculum size) and nutritional (medium compositions) factors. Optimal fermentation conditions for BLIS production were not necessarily appropriate for the good growth rate of *L.* *lactis* Gh1. The replacement of nitrogen and carbon sources to soytone and fructose as well as the mid-exponential age of inoculum at 1% (*v*/*v*) were the selected factors for high BLIS production by *L. lactis* Gh1. The modified BHI broth in the present work could represent an alternative medium for BLIS production since it permitted comparable BLIS level compared to conventional BHI broth. Results from this study could be used for subsequent application in process design and optimisation. In future, the integration of mathematical techniques such as response surface methodology (RSM) and artificial neural network (ANN) should be applied for systematic optimal medium formulation for BLIS production.

## Figures and Tables

**Table 1:** Composition of commercial BHI medium.

Component	Amount (g/L)
Nutrient substrate (Extract of brain and heart, and peptones)	27.5
Sodium chloride	5
Di-Sodium hydrogen phosphate	2.5
D (+) glucose	2

**Table 2:** Growth of *L. lactis* Gh1 and bacteriocin-like inhibitory substances (BLIS) production in various types of culture media.

Media	Time for P_mX_ (h)	pH		Maximum BLIS Activity P_mX_ (AU. mL^−1^)	Maximum Cell Concentration X_mX_ (g·L^−1^)	Specific Growth Rate μ_max_ (h^−1^)
		Initial	Final			
MRS	16	5.43 ± 0.01	4.27 ± 0.04	375.48 ± 35.43 ^b^	0.49 ± 0.002 ^a^	0.09 ± 0.0057 ^b^
M17	4	6.98 ± 0.01	6.49 ± 0.02	640.23 ± 21.06 ^a^	0.43 ± 0.020 ^b^	0.08 ± 0.0022 ^b^
BHI	4	6.95 ± 0.02	5.95 ± 0.00	672.86 ± 24.76 ^a^	0.46 ± 0.020 ^ab^	0.15 ± 0.0126 ^a^
TSB	4	6.92 ± 0.03	6.72 ± 0.02	637.84 ± 24.73 ^a^	0.48 ± 0.040 ^ab^	0.09 ± 0.0024 ^b^
LB	-	6.01 ± 0.01	3.66 ± 0.02	0.00 ± 0.00 ^c^	0.31 ± 0.002 ^c^	0.09 ± 0.0042 ^b^

Note: all values are expressed as means ± standard deviation in triplicate. Data followed by the same letters are not significantly different (*p* < 0.05) according to Duncan’s multiple range test to evaluate the effect of investigated parameters. S.D: standard deviation.

**Table 3:** Effects of initial culture pH on growth of *L. lactis* Gh1 and BLIS production using BHI medium.

Initial pH	Time for P_mX_ (h)	pH		Maximum BLIS Activity P_mX_ (AU. mL^−1^)	Maximum Cell Concentration X_mX_ (g·L^−1^)	Specific Growth Rate μ_max_ (h^−1^)
		Initial	Final			
2	-	2.10 ± 0.00	2.10 ± 0.00	0.00 ± 0.00 ^d^	0.00 ± 0.000 ^e^	0.000 ± 0.0000 ^c^
3	-	3.02 ± 0.00	3.02 ± 0.00	0.00 ± 0.00 ^d^	0.00 ± 0.000 ^e^	0.000 ± 0.0000 ^c^
4	8	4.13 ± 0.01	3.95 ± 0.01	464.42 ± 7.58 ^c^	0.09 ± 0.000 ^d^	0.016 ± 0.0000 ^c^
5	6	5.00 ± 0.04	4.48 ± 0.02	475.17 ± 5.45 ^c^	0.29 ± 0.002 ^b^	0.050 ± 0.0005 ^b^
6	6	6.37 ± 0.00	5.13 ± 0.02	548.67 ± 22.85 ^b^	0.30 ± 0.000 ^b^	0.051 ± 0.0003 ^b^
7.04 *	6	7.10 ± 0.05	6.37 ± 0.00	599.45 ± 11.77 ^a^	0.36 ± 0.013 ^a^	0.104 ± 0.0025 ^a^
8	6	7.66 ± 0.01	7.10 ± 0.02	581.99 ± 26.38 ^ab^	0.27 ± 0.022 ^b^	0.046 ± 0.0166 ^b^
9	6	8.54 ± 0.02	7.89 ± 0.01	572.28 ± 26.64 ^ab^	0.22 ± 0.020 ^c^	0.043 ± 0.0142 ^b^

Note: all values are expressed as means ± standard deviation in triplicate. Data followed by the same letters are not significantly different (*p* < 0.05) according to Duncan’s multiple range test to evaluate the effect of investigated parameters. S.D: standard deviation. * Control (BHI broth without pH adjustment).

**Table 4:** Growth of *L. lactis* Gh1 and BLIS production using BHI medium, inoculated with different ages of inoculum. The inoculum age was set at 1% (*v/v*).

Growth Phase	Time for P_mX_ (h)	pH		Maximum BLIS Activity P_mX_ (AU. mL^−1^)	Maximum Cell Concentration X_mX_ (g·L^−1^)	Specific Growth Rate μ_max_ (h^−1^)
		Initial	Final			
Mid-exponential	6	6.91 ± 0.01	6.31 ± 0.00	665.96 ± 1.83 ^a^	0.28 ± 0.033 ^a^	0.113 ± 0.010 ^a^
Late-exponential	6	6.90 ± 0.01	6.28 ± 0.01	615.73 ± 5.34 ^b^	0.33 ± 0.017 ^a^	0.110 ± 0.004 ^a^
Stationary	8	6.90 ± 0.02	6.33 ± 0.01	627.98 ± 16.74 ^b^	0.31 ± 0.033 ^a^	0.100 ± 0.020 ^a^

Note: all values are expressed as means ± standard deviation in triplicate. Data followed by the same letters are not significantly different (*p* < 0.05) according to Duncan’s multiple range test to evaluate the effect of investigated parameters. S.D: standard deviation.

**Table 5:** Growth of *L. lactis* Gh1 and BLIS production using BHI medium, inoculated with different sizes of inoculum. The inoculum was set at mid-exponential phase.

Inoculum Size % (*v*/*v*)	Time for P_mX_ (h)	pH		Maximum BLIS Activity P_mX_ (AU. mL^−1^)	Maximum Cell Concentration X_mX_ (g L^−1^)	Specific Growth Rate μ_max_ (h^−1^)
		Initial	Final			
0.5	8	7.15 ± 0.01	6.33 ± 0.00	593.02 ± 57.36 ^b^	0.33 ± 0.004 ^d^	0.071 ± 0.001 ^d^
1.0	6	7.13 ± 0.01	6.33 ± 0.01	700.24 ± 55.44 ^a^	0.34 ± 0.002 ^c^	0.071 ± 0.001 ^d^
2.0	6	7.10 ± 0.00	6.29 ± 0.02	538.32 ± 72.05 ^bc^	0.42 ± 0.002 ^b^	0.095 ± 0.003 ^c^
4.0	4	7.06 ± 0.01	6.25 ± 0.00	532.83 ± 7.69 ^bc^	0.44 ± 0.005 ^a^	0.151 ± 0.001 ^a^
6.0	4	7.03 ± 0.04	6.22 ± 0.04	535.39 ± 17.98 ^bc^	0.44 ± 0.004 ^ab^	0.096 ± 0.002 ^c^
8.0	8	7.00 ± 0.02	6.21 ± 0.03	566.08 ± 18.36 ^b^	0.43 ± 0.002 ^ab^	0.092 ± 0.010 ^c^
10.0	10	6.95 ± 0.01	6.18 ± 0.02	479.60 ± 32.10 ^c^	0.42 ± 0.016 ^ab^	0.112 ± 0.003 ^b^

Note: all values are expressed as means ± standard deviation in triplicate. Data followed by the same letters are not significantly different (*p* < 0.05) according to Duncan’s multiple range test to evaluate the effect of investigated parameters. S.D: standard deviation.

**Table 6:** Growth of *L. lactis* Gh1 and BLIS production using BHI medium, inoculated with different sub-culturing frequencies of inoculum. The inoculum was set at mid-exponential phase; at 1% (*v*/*v*) inoculum size.

Sub-Culturing Frequency	Time for P_mX_ (h)	pH		Maximum BLIS Activity P_mX_ (AU. mL^−1^)	Maximum Cell Concentration X_mX_ (g·L^−1^)	Specific Growth Rate μ_max_ (h^−1^)
		Initial	Final			
One time	10	7.15 ± 0.01	6.55 ± 0.01	553.45 ± 4.58	0.27 ± 0.004	0.05 ± 0.001
Two times	6	7.12 ± 0.01	6.49 ± 0.01	728.83 ± 37.80	0.21 ± 0.002	0.05 ± 0.001

**Table 7:** Growth of *L. lactis* Gh1 and BLIS production by using modified BHI medium supplemented with different organic nitrogen (N) sources. All nitrogen sources were added at concentration of 4.6 g/L N.

Nitrogen Source	Time for P_mX_ (h)	pH		Maximum BLIS Activity P_mX_ (AU. mL^−1^)	Maximum Cell Concentration X_mX_ (g·L^−1^)	Specific Growth Rate μ_max_ (h^−1^)
		Initial	Final			
BHI	6	6.94 ± 0.01	6.28 ± 0.00	567.05 ± 47.01 ^bc^	0.35 ± 0.004 ^d^	0.074 ± 0.0012 ^b^
No nitrogen sources	-	6.86 ± 0.00	6.29 ± 0.02	0.00 ± 0.00 ^e^	0.00 ± 0.00 ^i^	0.000 ± 0.0000 ^d^
**Organic Nitrogen**						
Yeast extract	4	6.46 ± 0.01	4.98 ± 0.01	619.41 ± 26.02 ^b^	0.45 ± 0.005 ^b^	0.107 ± 0.0040 ^c^
Meat extract	4	6.51 ± 0.02	5.38 ± 0.01	594.87 ± 8.69 ^b^	0.42 ± 0.011 ^c^	0.1062 ± 0.0050 ^a^
Peptone	4	6.82 ± 0.03	6.58 ± 0.00	580.00 ± 14.14 ^b^	0.36 ± 0.016 ^d^	0.0547 ± 0.0025 ^bc^
Soytone	4	6.80 ± 0.00	4.78 ± 0.01	764.71 ± 15.15 ^a^	0.69 ± 0.026 ^a^	0.0542 ± 0.0274 ^c^
Tryptone	4	7.02 ± 0.01	6.71 ± 0.02	515.59 ± 33.57 ^cd^	0.20 ± 0.001 ^e^	0.0429 ± 0.0005 ^c^
**Inorganic Nitrogen**						
(NH_4_)2SO_4_	6	6.86 ± 0.02	5.40 ± 0.00	599.08 ± 35.88 ^b^	0.07 ± 0.0013 ^f^	0.0120 ± 0.0004 ^d^
NH_6_PO_4_	6	5.08 ± 0.02	5.10 ± 0.01	509.07 ± 26.23 ^d^	0.01 ± 0.0007 h^i^	0.0007 ± 0.0005 ^d^
NH_4_NO_3_	-	6.82 ± 0.01	6.25 ± 0.01	0.00 ± 0.00 ^e^	0.03 ± 0.0004 ^gh^	0.0060 ± 0.0010 ^d^
NH_4_Cl	-	6.44 ± 0.00	5.98 ± 0.02	0.00 ± 0.00 ^e^	0.03 ± 0.0011 ^g^	0.0076 ± 0.0001 ^d^

Note: all values are expressed as means ± standard deviation in triplicate. Data followed by the same letters are not significantly different (*p* < 0.05) according to Duncan’s multiple range test to evaluate the effect of investigated parameters. S.D: standard deviation.

**Table 8:** Growth of *L. lactis* Gh1 and BLIS production by using modified BHI medium supplemented with 4.6 g/L N of soytone at different carbon sources. All carbon sources were added at 2 g/L.

Carbon Source	Time for P_mX_ (h)	pH		Maximum BLIS Activity P_mX_ (AU. mL^−1^)	Maximum Cell Concentration X_mX_ (g·L^−1^)	Specific Growth Rate μ_max_ (h^−1^)
		Initial	Final			
BHI	8	7.11 ± 0.00	6.19 ± 0.01	530.12 ± 3.38 ^d^	0.24 ± 0.0005 ^g^	0.061 ± 0.0003 ^f^
No carbon sources	10	6.89 ± 0.01	5.68 ± 0.00	634.82 ± 26.40 ^bc^	0.56 ± 0.0018 ^f^	0.046 ± 0.0003 ^g^
**Monosaccharides**						
Fructose	10	6.86 ± 0.01	4.96 ± 0.00	715.36 ± 13.77 ^a^	0.66 ± 0.0018 ^c^	0.108 ± 0.0007 ^e^
Glucose	10	6.79 ± 0.00	4.79 ± 0.02	674.03 ± 25.05 ^abc^	0.67 ± 0.0110 ^b^	0.139 ± 0.0066 ^c^
Galactose	10	6.86 ± 0.02	5.15 ± 0.02	678.51 ± 1.23 ^abc^	0.65 ± 0.0018 ^d^	0.214 ± 0.0011 ^a^
**Disaccharides**						
Sucrose	8	6.98 ± 0.00	4.85 ± 0.01	661.77 ± 17.54 ^abc^	0.70 ± 0.0018 ^a^	0.136 ± 0.0010 ^c^
Lactose	8	6.98 ± 0.00	5.53 ± 0.02	622.99 ± 28.62 ^c^	0.60 ± 0.0018 ^e^	0.123 ± 0.0018 ^d^
Maltose	8	6.99 ± 0.01	5.24 ± 0.02	687.45 ± 43.19 ^ab^	0.67 ± 0.0018 ^b^	0.198 ± 0.0010 ^b^
**Sugar Alcohol**						
Sorbitol	8	7.01 ± 0.01	5.68 ± 0.00	636.48 ± 21.62 ^bc^	0.56 ± 0.0018 ^f^	0.206 ± 0.0027 ^ab^
Mannitol	8	7.00 ± 0.02	5.16 ± 0.02	651.78 ± 9.69 ^bc^	0.57 ± 0.0037 ^f^	0.199 ± 0.0011 ^b^

Note: all values are expressed as means ± standard deviation in triplicate. Data followed by the same letters are not significantly different (*p* < 0.05) according to Duncan’s multiple range test to evaluate the effect of investigated parameters. S.D: standard deviation.
